# How decadal predictions entered the climate services arena: an example from the agriculture sector

**DOI:** 10.1016/j.cliser.2022.100303

**Published:** 2022-08

**Authors:** Balakrishnan Solaraju-Murali, Dragana Bojovic, Nube Gonzalez-Reviriego, Andria Nicodemou, Marta Terrado, Louis-Philippe Caron, Francisco J. Doblas-Reyes

**Affiliations:** a Barcelona Supercomputing Center (BSC), Carrer de Jordi Girona 29, 08034 Barcelona, Spain; b Ouranos, 550 Sherbrooke St W, Montreal, Quebec H3A 1B9, Canada; c Institució Catalana de Recerca i Estudis Avançats (ICREA), Passeig de Lluis Companys 23, 08010 Barcelona, Spain

**Keywords:** Decadal climate forecast, Near-term prediction, Coproduction, Food security

## Abstract

Predicting the variations in climate for the coming 1–10 years is of great interest for decision makers, as this time horizon coincides with the strategic planning of stakeholders from climate-vulnerable sectors such as agriculture. This study attempts to illustrate the potential value of decadal predictions in the development of climate services by establishing interactions and collaboration with stakeholders concerned with food production and security. Building on our experience from interacting with users and the increased understanding of their needs gathered over the years through our participation in various European activities and initiatives, we developed a decadal forecast product that provides tailored and user-friendly information about multi-year dry conditions for the coming five years over global wheat harvesting regions. This study revealed that the coproduction approach, where the interaction between the user and climate service provider is established at an early stage of forecast product development, is a fundamental step to successfully provide useful and ultimately actionable information to the interested stakeholders. The study also provides insights that shed light on the reasons for the delayed entry of decadal predictions in the climate services discourse and practice, obtained from surveying climate scientists and discussing with decadal prediction experts. Finally, it shows the key challenges that this new source of climate information still faces.

**Practical implications**:

The coproduction process is essential for developing useful forecast products and promoting the uptake of recently developed decadal climate information. In the context of climate services, coproduction starts with informing the relevant stakeholders about the potential role of climate information through different communication channels. It further involves knowledge exchange between scientists and users, as well as direct collaboration and co-design of new climate information, applying tools such as the development of case studies and user decision support systems. In this context, the present study strives to coproduce a climate service based on decadal predictions through the development of a tailored decadal forecast product in close collaboration with users from the agriculture sector, to demonstrate the potential applicability of such climate information.

Providing skillful and robust climate information on a multi-annual to decadal timescale holds the potential of being of great value for a broad range of users from different socio-economic sectors that are influenced by the varying climate conditions. In this study, through the interaction with stakeholders from the agriculture sector, we present some user needs and key decision areas that could be improved with decadal climate information. For instance, this type of climate information is useful for supporting planning decisions that require several years to be implemented, such as decisions in terms of equipment purchase (irrigation plants), planning of supply chain at international level and studying the use of new crop varieties. In addition, such information can have an impact on developing strategic policies related to agriculture, from regional to the EU common agricultural policy, as the policy changes within the agricultural sector usually happen at this timescale.

## 1 Introduction

Decadal climate predictions represent a source of near-term, understood to span from the following few years to a couple of decades into the future, climate change and variability information that has the potential to improve climate-related decisions in a wide range of socio-economic sectors, which are heavily influenced by climate variability and change (Bruno Soares and Dessai, 2014). The first attempt at producing this type of climate information was made in the framework of the EU-funded ENSEMBLES project, an integrated research project that ran from 2004 to 2009 (van der Linden and Mitchell, 2009). Since then, the field of decadal predictions has grown significantly, in part due to the large socio-economic interest generated by these predictions. Clear examples of the growing interest in this field of research are the inclusion of decadal predictions in the recent phases of the Coupled Model Intercomparison Project (namely CMIP5 and CMIP6), the production and publication of real-time decadal predictions (Smith et al., 2013, Kushnir et al., 2019), and a growing body of literature on potential applications of these forecasts (Reyers et al., 2015, Caron et al., 2018, Solaraju-Murali et al., 2021).

Despite the scientific progress achieved in this field (Doblas-Reyes et al., 2013, Goddard et al., 2013, Smith et al., 2020, Delgado-Torres et al., 2022), only limited effort has gone into effectively using near-term climate forecasts for adaptation and mitigation purposes. This is probably linked, at least partially, to the lack of practical applications illustrating how climate information at this timescale can bring value to decision-making. Recent efforts have started addressing this gap by showcasing the quality of this type of forecasts in predicting extreme climate events, such as drought and heat stress, using user-relevant climate indicators for the agriculture sector (Paxian et al., 2019, Solaraju-Murali et al., 2019, Solaraju-Murali et al., 2021). Such assessments pave the way to demonstrate the potential value that decadal predictions hold for agriculture.

While research to showcase the application of decadal predictions is rapidly evolving, the climate services community, which is working on enhancing the usability and facilitating the penetration of climate information in socio-economic sectors, is still barely using these predictions. Further efforts are needed to establish transdisciplinary partnerships between scientific and sectoral communities that would coproduce climate services based on decadal predictions and communicate the resulting climate information to a broader stakeholder community. This study aims to showcase an attempt made to coproduce decadal prediction-based climate services with users from the agriculture sector. At the same time, we wanted to shed light on the reasons behind the delayed arrival of decadal climate information into the climate services arena and the key challenges faced in this field. To do so, we opened a dialogue with the users to understand their perspective on the usability of decadal predictions, and a discussion with climate scientists to understand why it took longer for decadal predictions than for other sources of climate information to enter the climate services discourse and practice and what is novel about decadal predictions.

## 2 Near-term climate change information

**Fig. 1:**
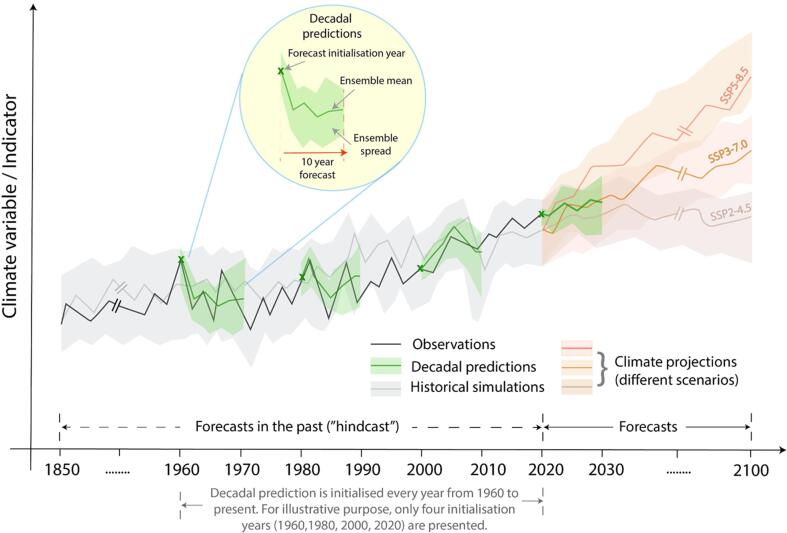
Illustration of the difference between decadal predictions and long-term climate change simulations. The black thick line represents the observations. The grey, green and tones of red correspond to the historical simulations, decadal predictions and climate projections under different socioeconomic scenarios, respectively. The thick lines show the ensemble mean and the shaded light colors are the ensemble spread, which indicates the uncertainty ranges associated with climate simulations.

Over the past few decades, the agriculture user community working on food production and security (such as the medium- to large-scale food companies, the Food and Agriculture Organization, and other relevant organisations) has been commonly using long-term climate change projections as an important source of climate change information (Bruno Soares et al., 2018). Only recently have decadal climate predictions been made available for users as an additional source of climate information. Even though the difference between decadal climate predictions and long-term climate change simulations is obvious for climate scientists, users are often not aware of such a distinction. In an attempt to enhance the understanding of the user community, Fig. 1 illustrates the difference between these two climate change information sources. The figure complements a number of schematic illustrations commonly used to illustrate such information in the literature (e.g., Meehl et al., 2009, Merryfield et al., 2020).

Climate models, which are a mathematical representation of the Earth’s climate and built using the basic laws of classical physics and thermodynamics, are used to run both long-term climate change simulations and decadal predictions. Climate change simulations are represented in Fig. 1 by both historical simulations (colored in grey), and climate projections (colored in tones of red). Historical simulations, where the climate model is typically run from the pre-industrial era (1850) to around present day, are prescribed with the best estimates of observed external factors that impact the climate, such as methane, carbon dioxide and ozone concentrations, solar radiation, aerosols from volcanic eruptions, aerosols from human activity, and land-use changes. Climate projections, in simple terms, are the extension of historical simulations wherein climate models are integrated under the influence of future greenhouse-gas emission scenarios derived from various Shared Socioeconomic Pathways (SSPs) from present day till the end of the twenty-first century. SSPs, such as SSP2-4.5 (CO2 emissions around current levels until 2050, then falling but not reaching net zero by 2100), SSP3-7.0 (CO2 emissions doubled by 2100 with respect to 2015 values) and SSP5-8.5 (CO2 emissions tripled by 2075 with respect to 2015 values), are the new range of scenarios developed to provide input to the latest climate models that are contributing to the Intergovernmental Panel on Climate Change sixth assessment report (IPCC, 2021). In the case of long-term climate change simulations, no information is provided to the model on the contemporaneous observed states of the Earth’s climate system (such as atmosphere, ocean, sea ice and land surface).

Decadal predictions (colored in green in Fig. 1) are produced with the same climate models as those used for the long-term climate change simulations, but are initialised by introducing the observed state of the climate system each year from 1960 to the near-present. The decadal predictions are run for 10 years under the influence of changing external forcings (for instance, with rising greenhouse-gas concentration). Initialisation is the process of phasing the model’s natural climate variability toward the observed climate state at the beginning of each prediction. For illustration purposes, we highlight decadal predictions for four initialisation years (1960, 1980, 2000, 2020) with an ‘X’ in Fig. 1. While some attempts have been made to provide forecasts prior to 1960, the ocean observational system is not deemed of sufficient quality at present to provide adequate initial conditions prior to that date (Müller et al., 2014).

In order to capture the uncertainties in climate simulations, ensembles of decadal predictions and climate projections are constructed by slightly varying the initial state of the climate system (in the case of the predictions, using an estimate of the observational uncertainty), by using a number of different models and/or by perturbing the parameters of a given climate model (Meehl et al., 2009). An individual prediction is known as an ‘ensemble member’, while the average of all the ensemble members is referred to as the ‘ensemble mean’ (represented with dark colored lines in Fig. 1). The color shadings around the means represent the ensemble spread that aims to capture the full prediction uncertainty linked to an incomplete knowledge of the initial conditions or to climate model errors.

It is fundamental to determine how well the climate model incorporates all the relevant components of the Earth’s climate system. For this reason, the climate community has traditionally compared the predictions of the past climate (‘hindcasts’) with the recorded observations. The forecasts are considered to be skillful if these hindcasts (i.e. the years corresponding to the dotted arrow in the bottom of Fig. 1) are able to capture the variations in the observed climate. Such an assessment helps to build confidence in the predictions and identify model limitations that should be addressed in future research. In addition to this, it is important to point out that inadequate representation of climate processes and their interactions with land, ocean, and the cryosphere in coupled climate models leads to systematic biases in the simulations. Therefore, the climate prediction community recommends using advanced correction techniques in order to deal with the biases and extract useful information from the raw output of the climate model (Bellprat et al., 2019).

## 3 Prototype decadal forecast product for the agriculture sector

Predicting the variations in climate for the upcoming 1–10 years is of great interest for decision makers, as this time horizon coincides with the strategic planning of many stakeholders (Bruno Soares et al., 2018). For this reason, a number of projects and initiatives have started to provide prototype climate services based on decadal climate predictions for different climate-vulnerable sectors such as infrastructure, energy, agriculture and insurance (Dunstone et al., 2022).

In an attempt to illustrate the potential value of decadal predictions in the development of climate services for the agriculture sector, we designed a prototype decadal forecast product that provides tailored, useful and user-friendly information for stakeholders in agriculture, in particular those from the wheat sector. In designing this prototype, we built on our experience interacting with users from the agriculture sector and the increased understanding of their needs gathered over the years through our participation in various EU-funded projects and initiatives (e.g. the MEDiterranean Grape, OLive and Durum wheat food systems - MED-GOLD and EUropean Climate Prediction - EUCP). The designed prototype was further developed under the framework of the Copernicus Climate Change Services (C3S; climate.copernicus.eu/sectoral-applications-decadal-predictions).

The prototype forecast product that was developed illustrates multi-annual predictions of drought conditions for the coming five years over global wheat harvesting regions. Based on previous interactions with users, the Standardised Precipitation Evapotranspiration Index (SPEI6) was selected to study the evolution of multi-year droughts. SPEI6 is the standardised estimate of climate water balance (i.e., the difference between monthly precipitation and potential evapotranspiration) accumulated over the six-month period prior to the wheat harvesting months over wheat harvesting areas. This prototype forecast product consists of a two-page document that presents a map of the most likely tercile category (below-normal (or dry), normal and above-normal (or wet) conditions estimated from the climatological distribution) of predicted SPEI6 for the period 2020–2024, as well as a time series of the historical values of predicted SPEI6 over a particular spatial grid point. It is followed by a brief text with the interpretation of the results, providing the users with an overview of the expected variation in the wet and dry conditions for the coming five years.

The prototype forecast product uses decadal forecasts (42 members in total) produced by four European institutions which update their forecast annually: 10 members from EC-Earth3 (Bilbao et al., 2021); 10 members from DePreSys4 (Sellar et al., 2020), 16 members from MPI-ESM1-2-LR (Müller et al., 2018) and 6 members from CMCC–CM2 (Cherchi et al., 2019). These are a set of 10-year long initialised forecasts that were simulated by explicitly prescribing the contemporaneous state of the climate system at the start of the simulation (November 1 of each year from 1960 to 2020), while also accounting for changes in radiative forcings (both natural and anthropogenic).

The document also provides the background information for obtaining the product along with a summary of the decadal forecast quality assessment. We use Ranked Probability Skill Score (RPSS; Wilks, 2006) and Relative Operating Characteristic (ROC) skill score (Broecker, 2011) to assess the forecast quality in predicting the SPEI6 at the multi-annual timescale. RPSS provides a measure of the skill of decadal forecasts in predicting the probabilities of the categorical events (tercile in our case) in comparison to that of an alternative reference forecast (the observed climatology in our case). On the other hand, the ROC skill score measures the ability of the decadal forecast system in discriminating the events and non-events that occurred in individual tercile categories, in comparison to the climatological forecast. Positive values of RPSS and ROC skill scores correspond to a skilful forecast compared to the reference forecast, whereas zero values correspond to a forecast that does not perform any better than the reference forecast. Negative values indicate that the forecast system performs worse than the reference.

The initial prototype forecast product is shown in Figs. S1 and S2 of supplementary material, along with the methodology used to produce this prototype forecast product in the Technical appendix section of the supplementary material. In this study, we present an evaluation of this initial prototype product and how it evolved and developed, through discussions and interactions with potential users, to match their needs.

## 4 Methodology

Given that the co-development of climate services based on decadal predictions is still in its early stage, this study aimed to better understand the current needs of stakeholders in the agriculture sector at this time scale and, at the same time, gather feedback on the user perception of the climate information provided in the forecast product introduced in Section 3.

For this purpose, we conducted an online workshop with stakeholders interested in multi-year drought predictions on 18th and 19th of February 2021. This workshop was organised as part of a C3S stakeholder event, whose main aim was to introduce general aspects of decadal predictions, challenges and opportunities to a wide range of stakeholders (>60 participants) and to showcase the usability of these predictions for decision-making in four particular socio-economic sectors (agriculture, infrastructure, insurance, and energy). This paper focuses on the interactions concerning the agriculture forecast product and the analysis of the conversations with stakeholders from the agrifood sector (11 participants).

The agriculture stakeholders were mainly interested in the applicability of the product to different crops (wheat, grape and cotton among others) and in food security. The prototype forecast product was sent to the participants ahead of the workshop for them to familiarise themselves with the forecast information. During the workshop, we facilitated the discussion by raising questions related to five different aspects: (i) opinion: whether decadal prediction would be useful for their institution and/or sector; (ii) clarity of content: whether the key concepts presented were easy to understand; (iii) clarity of format: whether the figures and presentation of information were clear; (iv) usefulness: whether the forecast information could be useful for their work and the work of others in their sector, and if modifications were needed; and (v) future applications: whether they saw potential future applications of decadal forecast product for both them and their sector.

After the workshop, the feedback provided by the participants on the forecast product was compiled and evaluated. For this, we used the coding technique supported by the qualitative analysis software MAXQDA, which organises the information by common topics and themes (Gibbs, 2007, Kuckartz and Rädiker, 2019). The forecast product was then revised to address the concerns raised during that workshop. The revised product sheet was shared via email with all the participants along with a short online questionnaire in July 2021. The aim of the questionnaire was to evaluate the clarity of the revised forecast product and its success in addressing the concerns and suggestions raised during the workshop. In addition, the questionnaire collected information on the need for further improvements, usability of the product and usefulness of this type of participatory activities.

In parallel to the evaluation of the decadal forecast product and its subsequent modifications driven by stakeholders’ feedback, this paper looks into the reasons, from a scientific perspective, as to why the use of decadal climate predictions were a latecomer in the climate services arena in comparison with other climate timescales. We conducted a short online survey to gain insight on the key challenges faced by the decadal prediction community, as well as to discuss the added value of using decadal predictions. The survey consisted of six questions and was completed by 13 climate scientists, among which 15% were senior scientists, 54% were mid-career researchers, 8% early-career researchers and 23% were anonymous. The participants had diverse experience in fundamental climate research such as model initialisation, ensemble generation, understanding the physical processes at the origin of predictability, bias adjustment, model evaluation and predicting extremes. In addition, they have contributed to the development of state-of-the-art climate forecast systems that predict climate at different timescales, ranging from a few weeks to a few decades into the future (sub-seasonal to decadal climate prediction).

The climate researchers were asked the following questions: (i) how they would explain decadal predictions to someone who is not a scientist; (ii) what makes decadal predictions different from climate information for other time scales; (iii) why research in decadal prediction has only started recently (roughly, in the last 15 years); (iv) what are the key challenges in making real-time decadal predictions; (v) whether there is an added value in using decadal predictions compared to climate projections; and (vi) for which sectors or applications decadal predictions could be useful.

The survey results were then further discussed in a roundtable discussion with decadal prediction experts, to find agreements and complement the expertise and experience of the authors. The answers obtained in the survey and the roundtable discussion were also analysed using the coding technique provided with the MAXQDA software.

## 5 Decadal prediction for climate services

### 5.1 Coproducing useful decadal climate information: the perspective of the agriculture sector

This section presents the outcomes of the workshop with stakeholders from the agriculture sector. The 11 workshop participants came from different agricultural sectors. This includes participants from: a family-owned wine company holding over 1500 hectares of vineyards; a retail firm that designs, manufactures and distributes clothing and sport goods; a company providing hi-tech solutions for sustainable management of crops such as wheat; and two public institutions directing its research capabilities to improve food security, as well as to help achieve climate and environmental goals. The discussion in the workshop addressed the participants’ perspective on the usability of decadal predictions in the agriculture sector and, more generally, identified a few key strengths and weaknesses of the developed decadal forecast product (described in Section 3).

#### 5.1.1 User needs for near-term climate information

Workshop participants were interested in decadal climate information as it covers a relevant temporal horizon for decision-making in the agriculture sector that is relatively unexplored at the moment.

One of the key benefits of decadal prediction, as recognized by participants from all the institutions, was that such information is important in supporting planning decisions that require several years to be implemented, such as decisions on crop plantation sites and crop varieties. “We need to make decisions like choosing the place where we are going to plant the crop, what varieties to use, if we need irrigation or not, and if we are bound to have a higher or lower consumption of water with time” (stakeholder from the wine sector). All these decisions can be well informed with both decadal predictions or long-term climate projections. Furthermore, decadal prediction could be a tool for improving the resilience of retail operations and the supply chain at the international level by predicting the climate extremes/shocks that could disrupt the global market. “We see the possibility of these predictions to help in developing business strategies at local and global levels by taking into account crop yield output scenarios of natural fibers under climate change conditions” (stakeholder from the retail sector).

Another example was provided by a participant working on food security research, who stated that multi-annual predictions can support their decadal outlook exercise, where they would potentially present the evolution of the global agriculture market in the coming ten years. In addition, “the policy changes within the agricultural sector usually happen at this timescale. For instance, the EU’s common agricultural policy (CAP) is acting on a 10-year timescale. Such an outlook, in terms of the economic component of the agriculture sector, is always looking at what could happen in the coming five to ten years by assessing the complex interaction between the biophysical, economic and climate components together, to understand what are the risks for the agriculture sector” (stakeholder working on food security research). Therefore, providing tailored information for the next five to ten years could provide robust information for policy makers from regional to national level, to better support the choices and the political agenda for the agriculture sector.

#### 5.1.2 Feedback and revision of the prototype decadal forecast product

During the workshop, a two-page prototype forecast product, described in Section 3, was showcased to the participants in order to gather feedback on the product. Here, we present the key concerns raised by the users on the first prototype product sheet (Supplementary Figs. S1 and S2) that was developed and a brief explanation of the changes made to the product sheet during the revision process to address those concerns.

The participants agreed that the concept and structure (format of the product sheet) was well drafted, but stated that the product sheet was targeting advanced stakeholders with technical experience as it contained climate-specific terms used by the forecasting community that might have different meanings within different stakeholder communities. For instance, a participant took notice of the usage of “normal”, “below-normal” and “above-normal” categories without clear definition in the prototype forecast sheet. “For forecasters, normal is a 30-year or multi-decadal average of data. On the contrary, for the common person, normal is something that is not unusual, which has a totally different meaning.” (stakeholder from the wine sector).

**Fig. 2:**
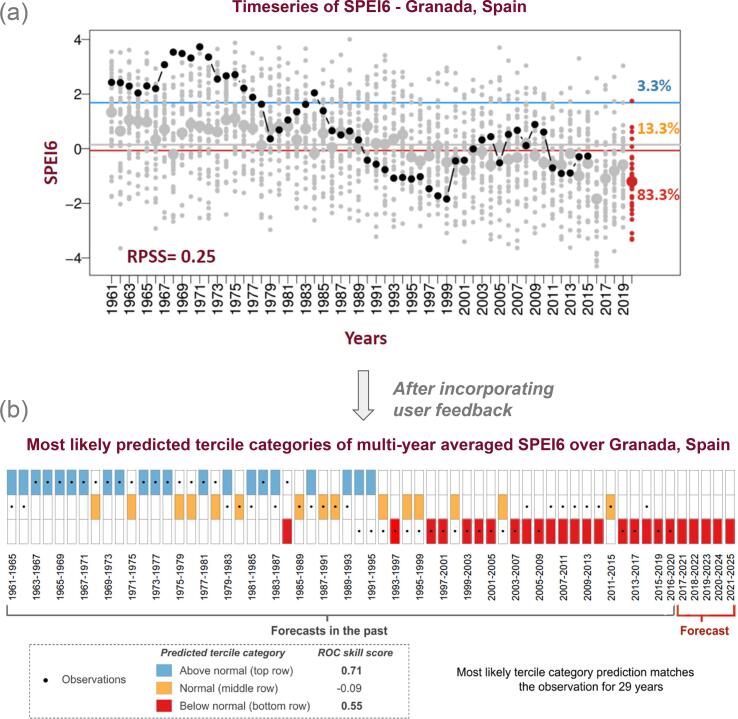
Changes made to the multi-annual averaged SPEI6 forecast time series plot by taking into account the user feedback. (a) The value of the predictions for each year corresponds to the SPEI6 predictions averaged over forecast years 1–5 during the wheat harvesting period over Granada, Spain. The small (large) grey dots correspond to the ensemble members (ensemble members mean) of the predictions issued for individual years. The red and blue horizontal lines show its lower and upper terciles of SPEI6, respectively. The black dots correspond to the observed SPEI6 values. The percentages indicate the fraction of members in each category, which is limited by the terciles. Skill score (RPSS) is shown in the lower part of the panel. (b) The multi-year averaged SPEI6 obtained with decadal prediction from 1961 to 2021 is displayed by a colored square: blue, yellow and red boxes indicate that the most likely tercile category of SPEI6 is above normal, normal and below normal, respectively. The black dots correspond to the category in which the observation falls. When the black dot falls on the red, yellow or blue box, the forecast matches the observation. The skill of the forecast system in predicting individual categories is presented with the ROC skill score (bold values represent skillful categories).

In addition, the participants found some of the information presented in the figures displayed in the prototype forecast product, such as the time series plot (Fig. 2a), hard to comprehend. “It takes time for someone to understand what the grey, black and red dots represent and then the information of the skill metric for probability forecasts (RPSS) further creates noise on understanding the information.” (stakeholder from the wine sector). Fig. 2a aimed to show how the users could benefit from regional or local scale climate information for farm-level crop management. The historical values of predicted SPEI6 over a specific point (Granada, Spain) is presented in Fig. 2a. The values of SPEI6 above the blue line and those below the red line correspond to periods of wetter and drier conditions, respectively. The small grey dots correspond to the ensemble members of the predictions issued for individual years and the large dots represent the average of all the ensemble members. The forecast issued in November 2019 shows that there is an 83.3% probability of dry conditions over Granada on average for the period 2020–2024, as 33 out of 40 ensemble members fall below the red line, which represents the lower tercile of SPEI6. The decadal forecast is found to perform better than the climatological distribution, as indicated by the positive skill (RPSS).

**Fig. 3:**
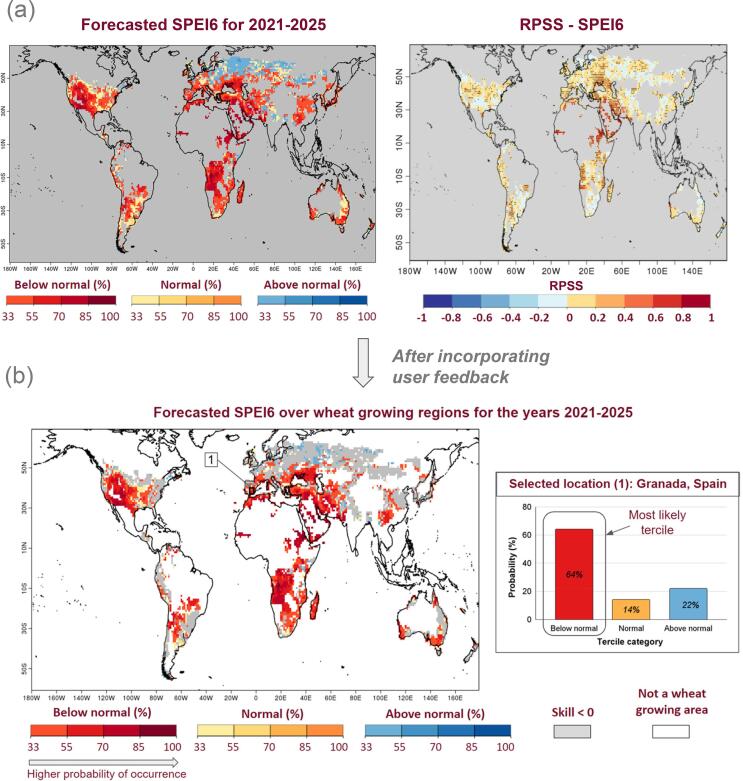
Changes made to the most likely tercile plot by taking into account the user feedback. (a) Most likely tercile category (below normal, normal and above normal) of SPEI6 over the global wheat harvesting regions is displayed on the left-side map, in which the colored grids show the category with the highest probability of occurrence. Ranked Probability Skill Score, RPSS of SPEI6 forecast averaged over years 1–5, during the wheat harvest month in each area, for the period 1961–2014 is presented in the right-side map. Most likely tercile category map in (b) is same as (a), but non-growing wheat areas and regions with negative skill (RPSS) are displayed in white and grey, respectively. The prediction of the selected grid-point (1 on the left-side map) is represented on the right-hand side of the figure where the probability of occurrence of each tercile category is shown for illustration.

According to feedback received, Fig. 2a was revised to better illustrate the time series information so that it would be clearer for both advanced users and users with less technical background. The new illustration displayed in Fig. 2b aims to show how the forecasts have performed in predicting the most likely category of SPEI6 in the past years and whether the prediction of a particular category is skilful. Concretely, Fig. 2b shows the most likely category of multi-annual averaged SPEI6 (boxes with red, yellow or blue colors) predicted over recent years for the selected area (grid box 1 in Fig. 3b), along with the corresponding observed category (black dots).

The decadal predictions discriminate between wet (above normal, blue boxes) and dry (below normal, red boxes) events relatively well, and the prediction of most likely category matched the observation for 29 years (corresponding to the total number of black dots in the red, yellow or blue boxes) out of 55 years. The relative operating characteristic (ROC) skill score metric was used to measure the ability of the forecast system to discriminate/predict a particular categorical event. The decadal predictions are found to have skill in predicting the above and below normal categories over the selected area, which is indicated by positive values of ROC skill score in Fig. 2b. A ROC skill score lower than 0 corresponds to a forecast with no skill, which is the case for the normal category.

Additionally, we have made an attempt to further simplify the global forecast map shown in Fig. 3a, in which the users had to compare the forecasted most likely tercile map (left side map of Fig. 3a) and the skill map (right side map of Fig. 3a) to know the regions that provide skillful forecasts. The below and above normal categories of SPEI6 in the left side map of Fig. 3a corresponds to periods of dry and wet conditions, respectively. After revision, the most likely tercile categories of SPEI6 for the wheat-growing regions that exhibit negative skill (RPSS<0) are masked in grey, while non-wheat growing regions are shown in white (Fig. 3b). The figure also illustrates how a particular most likely tercile category was chosen for each grid for clarity purposes. For instance, the most likely tercile category for the selected area in Fig. 3b (the closest grid point to Granada, Spain) is the below normal category with a 64% likelihood of occurrence and hence the chosen area is presented in red on the global map.

A workshop participant stated that it was unclear if the positive skill (RPSS>0) was an adequate baseline for risk assessment or for making decisions. For this reason, it would be important to offer users the opportunity to adjust the level of skill necessary to inform the decision, whether through the use of specific thresholds or a baseline to which the actual forecast can be compared to.

The participants thought that such global representation of forecast maps could mostly support strategic and infrastructure investments, or policy decisions. Along these lines a participant from the wine sector commented that “The global representation of forecast maps is mostly useful for large corporations and policy makers. It is less relevant for a [crop] grower or for someone working at a smaller scale. So, it would be important, considering the possibilities of having a higher resolution forecast for the region that someone can use to make decisions. Otherwise, you start leaving a lot of people out of the group of interest for this type of product“. Hence, it would be important to consider the possibility of creating decadal forecast products with a higher resolution for the region that small-scale food companies or farmers are interested in, to foster uptake of such information by the broader agriculture user community.

Finally, it was also mentioned that an important product for this timeframe and crop market, aside from the presented most likely tercile forecast of multi-annual averaged SPEI6, would be a product linked specifically to extremes. For instance, a participant working on food security research questioned: “Could you provide some information on extreme events? For instance, would the frequency of heat waves increase in the next 5 to 10 years”. Other participants were also interested in having the forecasts of growing season temperature and excess precipitation. Predicting the high or low probability of such climate events that are relevant for a particular crop would further encourage the users to adopt the decadal climate information in their decision-making process.

#### 5.1.3 Evaluation of the updated real-time forecast product

Following the product revision, we sent a short questionnaire to the workshop participants about the updated forecast product (Supplementary Figs. 3 and 4) that addressed the concerns raised during the workshop, and their responses are summarised in this section. In their evaluation of the updated forecast product, the users acknowledged the clarity and improvement of the product and confirmed that it successfully addressed much of the feedback provided in the workshop. The participants found the figures helpful in capturing the information. “Fig. 2b contains information for both experienced (with knowledge on what ROC is) and inexperienced users who can get an impression on forecast quality directly from the new visualisation” (stakeholder from the wheat sector).

When it came to the right amount of information, the opinion was split, which was not entirely surprising given the diversity of the user profiles and needs. One participant made further suggestions on how to improve and extend the background information section of the product, e.g. by detailing the wheat harvest months. Another participant, however, considered that the information “should be more summarised and condensed [for the general public].” (stakeholder from the wine sector). The same participant advocated for the use of stock images, e.g. of wheat fields, to improve visual attractiveness of the product. To address the food security concerns more generally, a participant suggested simultaneously showing forecasts for other major crops, such as maize, cotton and grape.

These key suggestions for further improvement will be addressed by continuing our collaboration with the participants in the future and tailoring the climate information based on their needs. Altogether, this particular decadal forecast product was perceived as useful for researchers and policy makers alike, in particular for the new European adaptation strategy and the CAP: “The information provided could serve to better foresee food security issues arising in the following 5 years, affecting market trading mechanisms, as well as allowing better preparation for grain yield redistribution.” (stakeholder working on food security research). Finally, the participation in the workshop and evaluation questionnaire was positively appraised by all the participants.

### 5.2 What is unique about decadal predictions: scientists’ perspective

In this section, we present results received from the survey sent to climate scientists, and a roundtable discussion with decadal prediction experts. The discussion on the perception about the position and late arrival of decadal predictions in the climate services arena was carried-out only with the climate scientists, as the aim of the survey was to understand the research challenges bound to this type of climate information.

According to the results from these interactions, climate scientists consider the novel aspect of decadal climate prediction to be that it takes into account the impact of changes in atmospheric composition and other external forcings, as well as the naturally generated internal climate variability. In particular, decadal predictions include both current or recent information about the climate state to initialise the climate model. Until the use of decadal predictions became more widespread, the time scale of 1–10 years was practically not covered in the production of climate information (Goddard et al., 2012), since seasonal predictions provide information up to one year ahead and climate projections are not “designed” to give meaningful climate information at this time horizon.

Evaluating the quality of the predictions is considered a fundamental step in climate prediction because it assesses whether the prediction system can be trusted to forecast certain events. This so-called verification process is typically based on validating extensive sets of hindcasts or retrospective predictions against observational references (Von and Zwiers, 2001). It takes an immense effort to produce such decadal hindcasts, which the roundtable participants saw as an obstacle. This is because long-term observational records of high quality and coverage, which are often not available beyond a few decades in the past, are needed for the initialisation of the climate model. In particular, having long observational records for the deep ocean is a challenge. Combining this with the computational power required to run a large set of predictions back in time (i.e., hindcasts are typically produced for the period 1961 to near-present) multiplies the amount of computational resources needed for decadal prediction. The round table participants suggested that these two aspects, namely long-term observational datasets and computational demand for producing decadal hindcasts, delayed developments of decadal prediction research compared to those seen for seasonal predictions or climate projections. Similarly, 70% of survey participants stated that computational resources were the principal limitation for the research on decadal prediction (Supplementary Fig. 6). The advancements in high-performance computing made these computationally heavy experiments possible in recent years.

Another aspect that postponed the development of decadal predictions, according to the roundtable and survey participants, is that there are few sources of decadal predictability in comparison to other timescales. Most of these sources of predictability, such as Atlantic Multi-decadal Variability (AMV; Ruprich-Robert et al., 2017) and Pacific Decadal Oscillation (PDO; Newman et al., 2016), come from the ocean. Transferring the predictability from the ocean to the atmosphere and over the continents has been yet another challenge during the development of decadal prediction. For example, there is a source of predictability in the North Atlantic Ocean, but “the North Atlantic is a very complex system with many interrelated processes in place and the current model resolution is not resolving all the relevant processes” (roundtable participant).

The roundtable discussion also considered the fact that decadal predictions had to learn both from the climate projection and seasonal prediction “schools of thought”, which could have limited advancements in this field. Finally, answering to the question why decadal predictions appeared as a latecomer in the climate information world, the climate scientists participating in the survey listed a few other factors, such the lack of an established “protocol” for developing and comparing decadal predictions and the lack of application (Supplementary Fig. 6).

As the main persisting challenge in making real-time decadal predictions, climate scientists perceived the lack of long homogenous past data series (Supplementary Fig. 7). As explained by one roundtable participant: “We could assimilate better observations, they are there, but they are not available for the years prior to the 1970s. It leaves you with fewer possibilities than in seasonal predictions. In seasonal prediction, you can focus only on the last 20 to 30 years for which you have satellite data. So there is a lack of flexibility [for model improvements through assimilation] in decadal predictions”. In addition, to introduce any improvements in the forecast system, the whole experiment needs to be redone. Hence, given the computational requirements of these experiments, scientists have a limited capacity to perform these experimental tests. “If we want to see what the impact of assimilating a particular variable is, we would need [to run in parallel] two decadal prediction experiments so that we can compare them, but it is too expensive. The high computational cost precludes you from doing tests” (roundtable participant).

Even though experimenting at the decadal timescale is limited due to high computational costs, investigating predictability at this time horizon brought several interesting insights to scientists: they learnt about the behaviour of some patterns that were not obvious before, for e.g. AMV has been linked to the observed slowdown of global warming over 1998–2012 through its impact on the tropical Pacific (Ruprich-Robert et al., 2021). In addition, according to the roundtable participants, experiments with decadal predictions taught scientists about the limits of predictability and to recognize that certain variables or regions are not predictable at certain time scales.

Evaluating decadal prediction has also allowed scientists to better understand the issues and errors present in climate models. “A prominent example is the signal-to-noise paradox (presented in Smith et al., 2020). Decadal predictions were where this concept was first established. This is one of the major challenges that the climate community is trying to deal with” (roundtable participant). The paradox is that the model is able to predict the real observed values better than it can predict its own simulated member, even with the underlying model deficiencies. Such an effect has now been widely documented in other experiments (Scaife and Smith, 2018, Siegert et al., 2016, Zhang and Kirtman, 2019), and the models are likely to underestimate decadal predictability in the regions that are impacted by the signal-to-noise paradox.

Finally, both the results from the survey and from the roundtable discussion showed that scientists perceived the narrowing of uncertainty about climate in the next 1 to 10 years as the key added value for society (Supplementary Fig. 8). It is expected that decadal predictions could help to reduce this uncertainty. Since developments are in progress, an interesting observation was that “decadal predictions may not yet have reached their full potential, due to issues related to the initialisation and model errors. Meaning that the added value may become clearer after further development” (survey participant).

## 6 Discussion

The work described in this paper aims to shed light on the reasons for the delayed entry of decadal predictions in the climate services discourse and practice. This was discussed with the scientific community which also helped us understand the key challenges that this new source of climate information still faces. As the climate services field lays at the user-provider interface, we put special emphasis in understanding how the information on decadal predictions should be coproduced and conveyed to stakeholders in order to enhance its usability. This insight was obtained in an interactive workshop with stakeholders from the agriculture sector, where the prototype decadal prediction product was presented and its improvements co-explored. Users evaluated the updated product in a follow-up survey, helping us co-design the final product. This aligns with the WMO guidance on good practices for climate services, which recommends the providers to have mechanisms in place for monitoring and evaluating their products (Adams et al., 2015).

The interaction with stakeholders from the agriculture sector helped us to link their expectations with the actual capabilities and limitations of decadal forecast systems. Addressing the user’s needs proved to be a challenging task requiring lasting collaboration and mutual understanding between scientists and users. For instance, the workshop participants expressed an interest in having high resolution forecasts for decision making. While such an exercise is possible and currently an ongoing research effort, it is a costly endeavour in terms of computing resources. A primary reason for the computational cost is the long period over which decadal prediction needs to be produced. One can estimate the computational cost associated with a single decadal forecast system running 10 year-long predictions once a year over the period 1961–2021 with 10 ensemble members as 61 start dates (1961–2021) x 10 forecast years x 10 ensemble members  = 6,100 simulated years. For the same ensemble size, such an experiment is more than twice as expensive as a typical historical and future scenario CMIP6 experiment, which covers the period 1850–2100, as pointed out during the roundtable discussion conducted in our study. Producing decadal predictions at higher resolution could be one to two orders of magnitude more expensive than running the prediction provided by the current state-of-the-art decadal forecast systems.

The SPEI forecast product delivered in the prototype was presented using terciles. This format, co-developed with users, aimed to showcase multi-year dry, normal, and wet conditions. However, users are not only interested in multi-annual prediction of the most likely tercile category of SPEI6 (Fig. 2, Fig. 3), but also in predicting the extremes (i.e., the highest and lowest values of the distribution), for instance. Moreover, there is a lot of room for tailoring the forecast of this index to their specific needs, based on a range of dry and wet conditions of interest. Thus, if we look at dry conditions, SPEI6 values go from normal (0 < SPEI6 < −0.8) to moderate (-0.80 < SPEI6 < −1.29) to severe (-1.30 < SPEI6 < −1.59) to extreme (-1.60 < SPEI6 < −1.99) to exceptional drought (SPEI6 < −2.0). While significant progress has been made in analysing extreme events in seasonal predictions (Hamilton et al., 2012, Bhend et al., 2017, Prodhomme et al., 2021) and climate projections (Russo and Sterl, 2011, Dosio, 2017, Seneviratne and Hauser, 2020), much less attention has been given to the evaluation of extremes in decadal predictions. A few studies have recently assessed the skill of decadal predictions at predicting extreme heat stress, cold and wet rainfall events (Eade et al., 2012, Hanlon et al., 2015, Solaraju-Murali et al., 2021). These studies generally found significant skill for extremes related to temperature and agreed that a substantial portion of that skill is linked to the long-term warming trend associated with the increase in anthropogenic greenhouse gases and aerosols, with the relative influence of natural variability and external forcings on forecast skill varying with the region considered.

During the interaction with stakeholders we also found that users in the agriculture sector traditionally based their decisions on deterministic information and that probabilistic forecasts were relatively new to them. This could be a reason why climate scientists find it challenging to communicate probabilistic information to users. Presenting technical concepts, such as the skill of probability forecasts in an intuitive way can favour the uptake of decadal climate information. Efforts to better link forecast quality and risks involved in making decisions based on probabilistic forecasts include tools and applications like the Weather Roulette app (Caron et al., 2018, Terrado et al., 2019) or the S2S4E Decision Support Tool (https://s2s4e-dst.bsc.es/).

Potential users of climate information are looking for the actual benefit that the skill information brings during their decision making process. While a number of studies have shown that decadal prediction can skillfully predict essential climate variables (van Oldenborgh et al., 2012, Corti et al., 2012, Doblas-Reyes et al., 2013) and user-relevant indices (Eade et al., 2012, Hanlon et al., 2015, Caron et al., 2015, Paxian et al., 2019, Solaraju-Murali et al., 2019, Solaraju-Murali et al., 2021), it is still unclear what skill level is adequate for each user to confidently use such climate information for decision making. To address this, the coproduction effort, including ongoing conversations between the climate scientists and users and close collaboration, is required, in order, for example, to co-explore and define a suitable skill threshold.

Users demand simple and intuitive visualisations, especially if the objective is that anyone (i.e., different user profiles in the agriculture sector, from farmers to field technicians, and from large food companies to food security policy makers) can understand and use the prediction (Calvo et al., 2022, Terrado et al., 2021). Also, different users from the agriculture sector require varied levels of information to be presented based on their needs. This would require the scientific community to sacrifice some technical detail in favour of a better understanding. Most of the time, users do not need access to all the available information, as having too much information can be overwhelming and make decision-making more challenging. Therefore, the visualisation needs to contain the right amount of information for the decision at hand, no less and no more.

This fine balance between simple and sufficiently informative could only be achieved in the process of co-exploring and co-designing climate information and decadal products between scientists and users. The coproduction approach applied in the current study confirmed what has been argued in the literature (e.g., Bojovic et al., 2019, Goddard et al., 2014, Bojovic et al., 2021), i.e., that engaging users at an early stage of the prototype development helped the co-design process and development of an advanced product that addressed users concerns and improved the product’s usability. Based on the knowledge exchange between scientists and users, the advanced product improved the presentation of probabilistic information and helped simplify visualisation and vocabulary, and fine-tune the amount of technical information presented in the product.

The collaboration with the 11 users from the various domains of the agriculture sector included an intensive interaction with climate service providers at the stakeholder workshop. The users had received the product draft prior to the workshop to familiarise themselves with the provided prototype forecast product. This setting helped the interaction between scientists (service providers) and the workshop participants (potential users) and allowed for building relationships. Ideally this type of collaboration could involve even more participants, to cover the array of user needs and expectations. However, keeping in mind that participants volunteer their time in these types of interactions (Turnhout et al., 2020), we could not expect these numbers to be as high as those achieved in low level engagement activities, such as consultations and surveys (Máñez Costa et al., 2022).

From the interaction with stakeholders, it is evident that there is an increasing interest in the decadal prediction. As well, there is an increasing interest within the climate services community in operationalization of climate prediction to support the uptake of such climate information by the user community. In this regard, there are already a number of initiatives being developed. As an example, the Grand Challenge on Near-Term Climate Prediction (GC-NTCP) aims to facilitate the development of decadal prediction towards its operational use. A primary goal of the GC-NTCP is to produce annually updated climate outlooks for the forthcoming years based on real-time forecasts produced by a number of institutions gathering both meteorological services and research institutes as part of an informal exchange (Smith et al., 2013). While the initiative has currently focussed on providing the forecasts of essential climate variables and indices, more user-focused prototypes of climate services for different sectors (agriculture, infrastructure, insurance and energy) have been explored by the Copernicus Climate Change Services (https://climate.copernicus.eu/sectoral-applications-decadal-predictions). Although the research about climate services on decadal timescale has just started to emerge and further research is still needed, this study provides evidence on the potential value that decadal predictions hold in supporting decision making and strengthening the resilience of climate-vulnerable sectors.

## 7 Conclusions

The present study evaluated the coproduction of a prototype climate service based on decadal predictions, which took place through interactions and collaboration between climate service providers and stakeholders from the agriculture sector participating in a number of European activities such as the MEDGOLD and EUCP projects, as well as C3S. As a part of the coproduction process, we developed a decadal forecast product that presents the multi-year forecast of SPEI6 for the coming five years over global wheat harvesting regions. The study summarises the lessons learnt from the interactions with the user community about their perspective on the usability of decadal predictions, and their feedback on the developed forecast product. The stakeholders expressed interest in uptaking decadal climate information as it presents potential in supporting planning decisions that take a number of years to develop or implement. For instance, the stakeholders interested in decadal prediction pointed out that this type of climate information could help to choose crop varieties or future crop plantation sites, to develop national policies such as the EU common agricultural policy and to develop business strategies at an international level.

The paper also presents the results received from the survey sent to climate scientists, and a roundtable discussion with decadal prediction experts. These interactions aimed to explore the perception of climate scientists on the reasons behind the late arrival of decadal predictions to the climate services arena and the key challenges faced by this research line. The participants considered that the key factors contributing to the delay in the development of decadal predictions are the following: the high computational demand for producing decadal hindcasts, the requirement of long homogeneous observational dataset for initialising the climate model and fewer sources of decadal predictability in comparison to other timescales. Nevertheless, since decadal predictions were first made available, the research line has been rapidly evolving. For instance, research has already started to generate seamless climate data sources from the near-term to longer timescales. The H2020 project EUCP is currently exploring the added value of blending together initialised decadal climate predictions with climate projections over the time period of 1 to 30 years (i.e., up to 2045), taking advantage of both approaches to provide a seamless multi-decadal climate information (Befort et al., 2020, Mahmood et al., 2021). In addition, other research initiatives are currently underway to improve decadal climate prediction systems (Kushnir et al., 2019, Merryfield et al., 2020).

While such advances in research aims to further enhance the predictive skill and reliability of available climate information, the development of climate services based on decadal predictions is still in its early stages. Tailoring near-term climate information to different socio-economic sectors, through building partnerships between the climate service providers and the user community, is an important first step for developing successful climate services. Therefore, identifying and engaging with potential users, and communicating the value of using near-time climate information during their decision-making processes should be considered as the utmost priority for ensuring the uptake and usage of this new source of climate information.

## CRediT authorship contribution statement

**Balakrishnan Solaraju-Murali:** Conceptualization, Formal analysis, Writing - original draft, Visualization. **Dragana Bojovic:** Conceptualization, Formal analysis, Writing - review & editing, Supervision. **Nube Gonzalez-Reviriego:** Conceptualization, Writing - review & editing, Supervision, Funding acquisition. **Andria Nicodemou:** Conceptualization, Writing - review & editing. **Marta Terrado:** Conceptualization, Writing - review & editing. **Louis-Philippe Caron:** Writing - review & editing, Supervision. **Francisco J. Doblas-Reyes:** Writing - review & editing, Funding acquisition.

## Declaration of Competing Interest

The authors declare that they have no known competing financial interests or personal relationships that could have appeared to influence the work reported in this paper.
