# Supplementary material for: How decadal predictions entered the climate services arena: an example from the agriculture sector
Source: Clim Serv. 2022 Aug;27:100303. doi: 10.1016/j.cliser.2022.100303 (PMC9380416; doi:10.1016/j.cliser.2022.100303)
Supplement: Supplementary data 1 [file mmc1.docx]

**Supplementary material for**

**How decadal predictions entered the climate services arena: an example from the agriculture sector**

Balakrishnan Solaraju-Murali^1,*^, Dragana Bojovic^1^, Nube Gonzalez-Reviriego^1^, Andria Nicodemou^1^, Marta Terrado^1^, Louis-Philippe Caron^1,2^ and Francisco J. Doblas-Reyes^1,3^

^1^ Barcelona Supercomputing Center (BSC), Carrer de Jordi Girona 29, 08034, Barcelona, Spain

^2^ Ouranos, 550 Sherbrooke St W, Montreal, Quebec H3A 1B9, Canada

^3^ Institució Catalana de Recerca i Estudis Avançats (ICREA), Passeig de Lluis Companys 23, 08010, Barcelona, Spain

* balakrishnan.solaraju@bsc.es


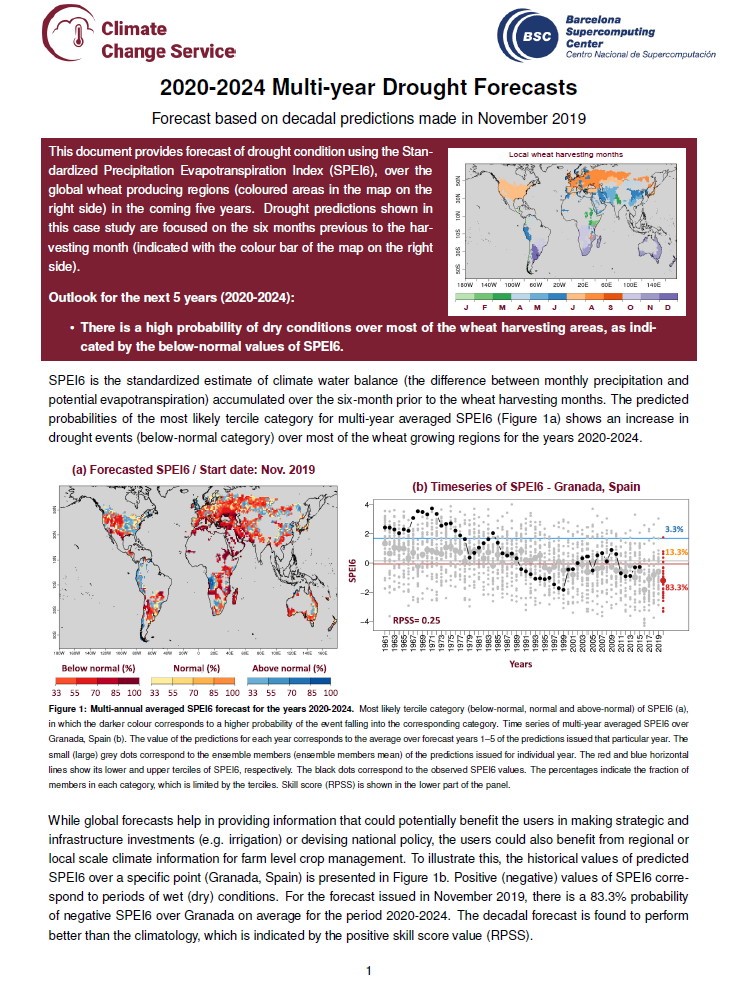


**Figure S1: Initial prototype product sheet** Page 1 of prototype forecast product for the years 2020-2024


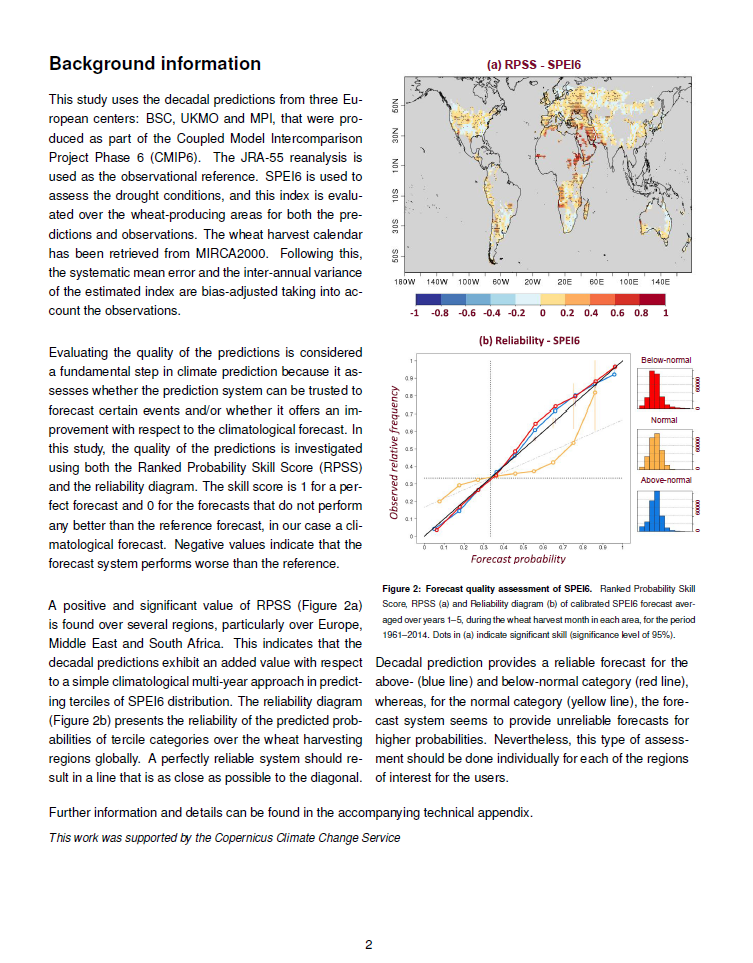


**Figure S2: Initial prototype product sheet** Page 2 of prototype forecast product for the years 2020-2024


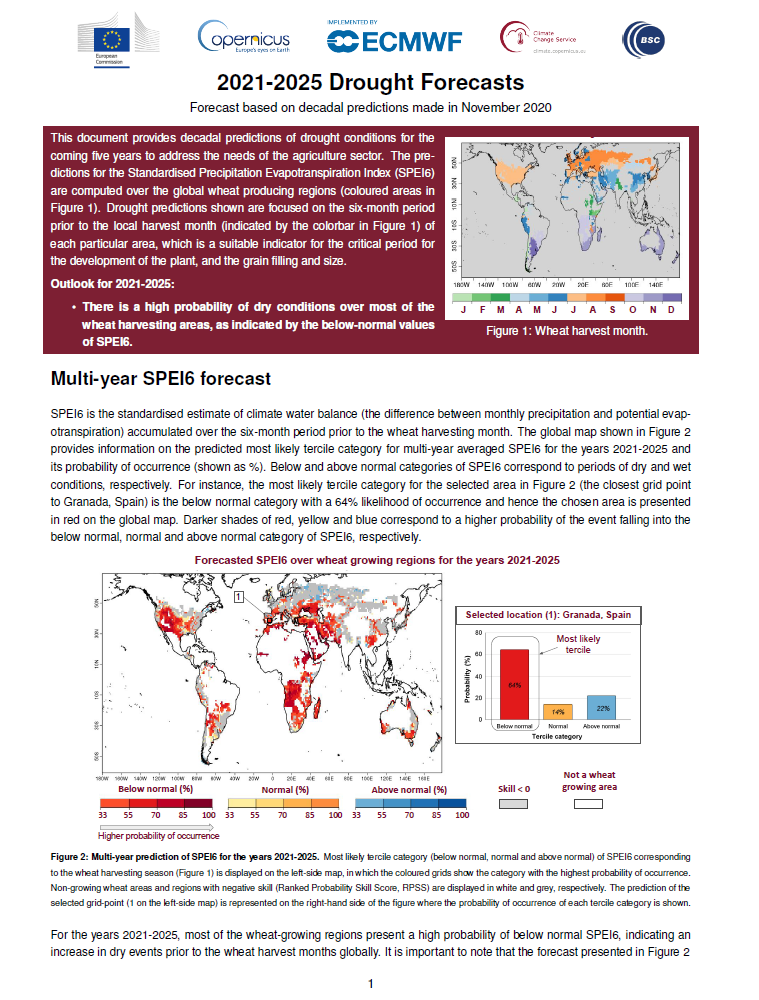


**Figure S3:**  **Revised real-time product sheet** Page 1 of real-time forecast product for the years 2021-2025. This product has been updated taking into account the feedback provided by workshop participants on the prototype (Figure S1 and S2).


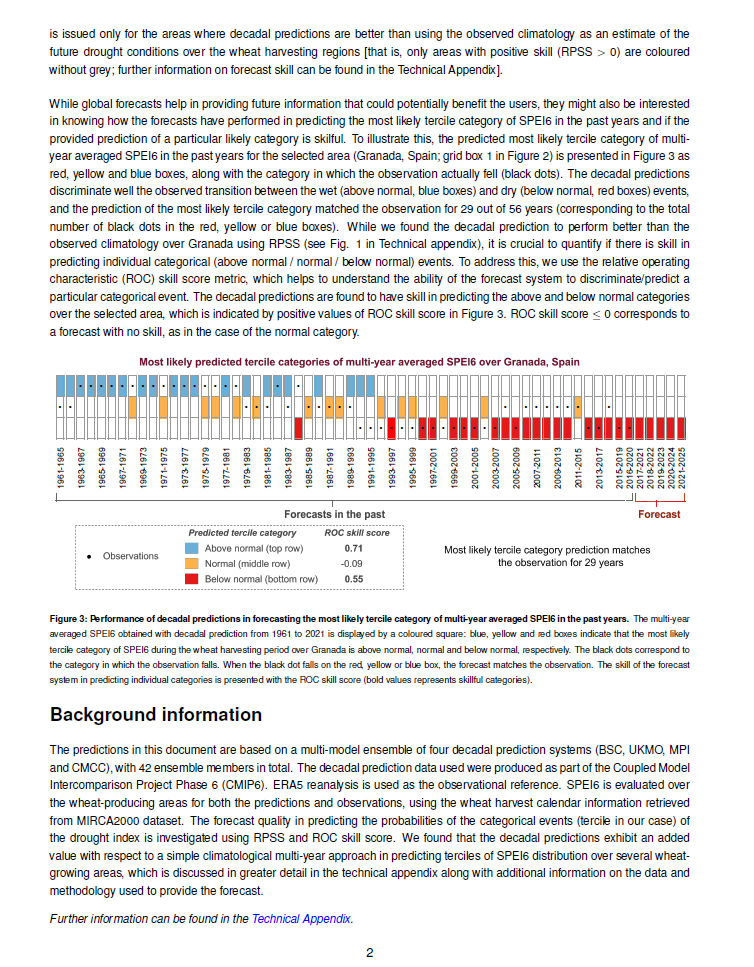


**Figure S4:** **Revised real-time product sheet** Page 2 of real-time forecast product for the years 2021-2025. This product has been updated taking into account the feedback provided by workshop participants on the prototype (Figure S1 and S2)

# **Technical appendix for the forecast product of the agriculture case study**

## **Model and observation data**

In this study we use four decadal prediction systems (42 members in total): 10 members from EC-Earth3 (Bilbao et al., 2021); 10 members from DePreSys4 (Sellar et al., 2020), 16 members from MPI-ESM1-2-HR (Müller et al., 2018) and 6 members from CMCC-CM2 (Cherchi et al. 2018). The considered models were run by explicitly prescribing the contemporaneous state of the climate system at the start of the simulation (November 1 of each year from 1960 to 2020), while also accounting for changes in radiative forcings (both natural and anthropogenic). For forecast quality assessment of agro-climatic indices, we use ERA5 (Hersbach et al., 2020) as a reference dataset. This dataset is selected due to its temporal and geographical coverage and spatial resolution and has been used to obtain the monthly temperature and precipitation globally for SPEI estimation.

The information of the local wheat harvesting month is retrieved from the MIRCA2000 dataset (Portman et al., 2010). In order to compare the datasets, the values of the climate variables and the wheat harvesting month are interpolated using the first-order conservative remapping approach, from their original grid to a grid with nominal 1° spatial resolution.

## **Post-processing and evaluation methods**

We have assessed the skill of the climate model at forecasting drought conditions over the wheat harvesting region for the forecast years 1 to 5. The drought conditions are estimated using the Standardised Precipitation Evapotranspiration Index aggregated over six months (SPEI6; Vicente-Serrano et al., 2010) index. The computed index is bias-adjusted using the calibration approach presented in Doblas-Reyes et al. (2005). After the calibration, we construct a large multi-model ensemble by pooling all the members of each individual forecast system together. The detailed methodology is presented in Solaraju-Murali et al., 2021.

The skill scores based on Ranked probability skill (RPS) and relative operating characteristic (ROC) skill are used to assess the forecast quality of predicted probabilities of tercile categories by decadal forecasts. RPS is the squared distance between the cumulative probabilities of the categorical forecast (tercile events in our case) and its corresponding observational reference. On the other hand, the ROC skill relates the hit rate to the corresponding false-alarm rate of a particular categorical event. For instance, while assessing the above normal temperature events, the hit rate is considered as the fraction of the observed above normal events that were correctly forecasted, whereas the corresponding false-alarm rate is the fraction of the predicted above normal events that did not occur in reality (i.e., were false alarms). This skill measure helps to understand the ability of the forecast system to discriminate between events and non-events of a particular categorical event, in general.

Often, it is of interest to evaluate forecasts with respect to a baseline. This baseline can either be a simpler and/or cheaper alternative (e.g. climatology) or a previous version of the forecast system. Such assessment provides the user with information on the added value of the decadal prediction system against an alternative approach. Therefore, the skill measures are reformulated as skill scores (RPSS, ROC skill score) by comparing the skill obtained from the decadal forecasts to the corresponding skill obtained from a reference forecast, in our case a climatological forecast. Positive values of RPSS and ROC skill scores correspond to a skilful forecast compared to the reference forecast, whereas zero values correspond to a forecast that does not perform any better than the reference forecast. Negative values indicate that the forecast system performs worse than the reference.

## **Analysis protocol for forecast product**

- Preparation of model and observational data:

Interpolated the essential climate variables from their original grid to a grid with nominal 1° spatial resolution for all the considered forecast systems and the reference dataset.

- Estimation of monthly climate water balance:

Computed the potential evapotranspiration (PET) and monthly climatic water balance (defined as the difference between monthly precipitation and PET). In our case, PET is estimated using the classical temperature-based Thornthwaite method (Thornthwaite 1948), owing to its simplicity and limited data requirements.

- Six-month accumulation of the estimated climate water balance:

For each grid, the wheat harvesting month information is gathered and the monthly climate water balance values are summed over six months prior to the wheat harvesting month (inclusive) for individual years. For example, the accumulated value for the region with wheat harvest in June is obtained as the sum of January to June climate water balance values for a specific year.

- Multi-annual averaging of the accumulated values:

The accumulated values are then averaged over five years for each harvest month. For example, the multi-annual average of accumulated climate water balance value (forecast years 1–5) for the month of June for the forecast initialised in November 1960 is the average of the 1961–1965 June accumulated values, and for the forecast initialised on November 1961, the average is obtained with the 1962–1966 June accumulated values, and so on.

- Estimation of multi-annual SPEI6:

The multi-annual averaged accumulated values are then fitted to a suitable parametric probability distribution and standardised to obtain SPEI6. Three-parameter shifted log-logistic distribution is used to fit the climate water balance values for SPEI6 in our case. We use the entire hindcast period (1961-2015) as a reference for standardisation.

- Calibration and multi-model combination of SPEI6:

The computed index of the individual forecast system is calibrated. Following this, a large multi-model ensemble is constructed by pooling individual forecast system members together.

- Calculation of skill scores of the probabilistic decadal forecast
- Display of the results:

The predicted likelihood map (in %) of the most likely tercile categories over winter wheat harvesting regions along with the illustration of the performance of decadal predictions in forecasting the most likely category of multi-year averaged SPEI6 in the past years over Granada, Spain are presented.

## **Forecast skill of SPEI6**

Evaluating the quality of the predictions is considered a fundamental step in climate prediction because it assesses whether the prediction system can be trusted to forecast certain event categories and/or whether it offers an improvement with respect to the climatological forecast. The quality of the predictions over global wheat harvesting regions are investigated using the RPSS. We have used the hindcast period (1961-2015) as a reference for climatological forecast.

Figure S5 shows the RPSS applied in the forecast product. A positive and significant value of RPSS is found over several regions, particularly over the Mediterranean, Middle East, and South Africa. This indicates that the decadal predictions exhibit an added value with respect to a simple climatological multi-year approach in predicting terciles of SPEI6 distribution. The forecast is presented only for the areas that exhibit positive skill.


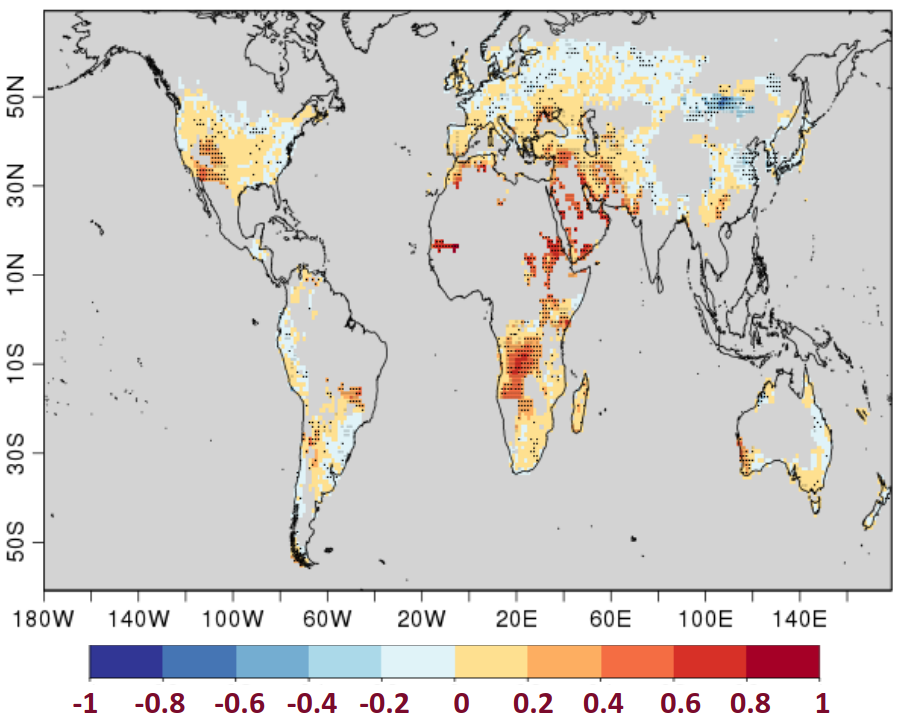


**Figure S5: Forecast quality assessment of SPEI6.** Ranked Probability Skill Score (RPSS) of calibrated SPEI6 forecast averaged over years 1–5 during the wheat harvest month in each area (presented in Fig. 1 of the forecast product) for the period 1961–2014. Dots indicate the areas where decadal predictions provide significantly better forecasts than the reference forecast at the 95% confidence level based on a random walk test (DelSole and Tippett, 2016) which checks whether the simulations outperform the reference forecast during a significant number of years. The areas over which the wheat is not harvested is represented in grey.

# **Generalised analysis of survey responses from climate scientists**


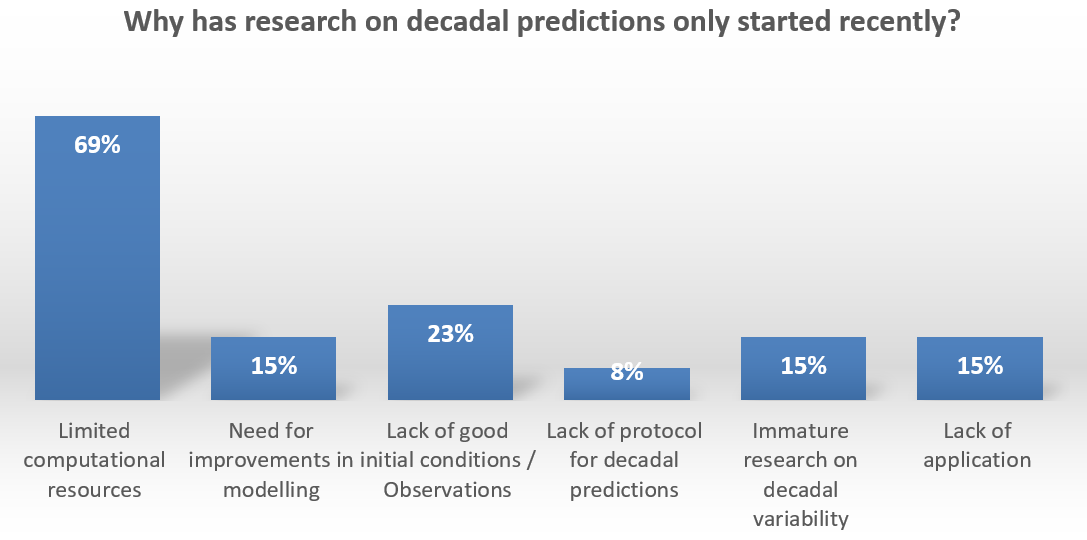


**Figure S6:** Aggregated survey response from the climate scientists on ‘why has research on decadal predictions only started recently?’


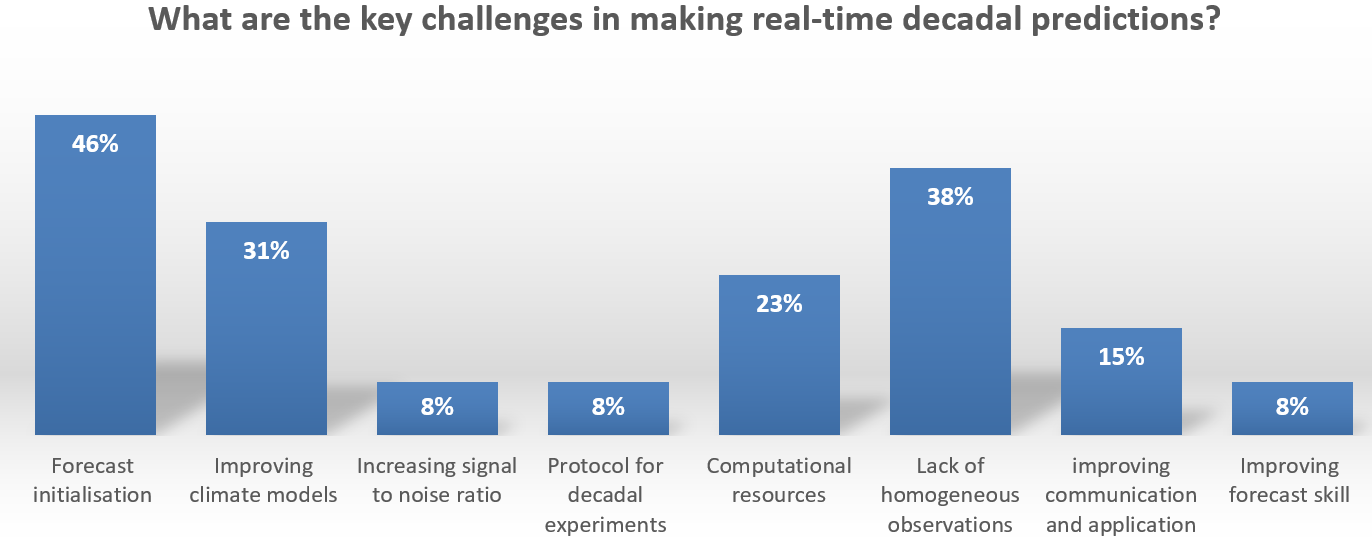


**Figure S7:** Aggregated survey response from the climate scientists on ‘what are the key challenges in making real-time decadal prediction?’


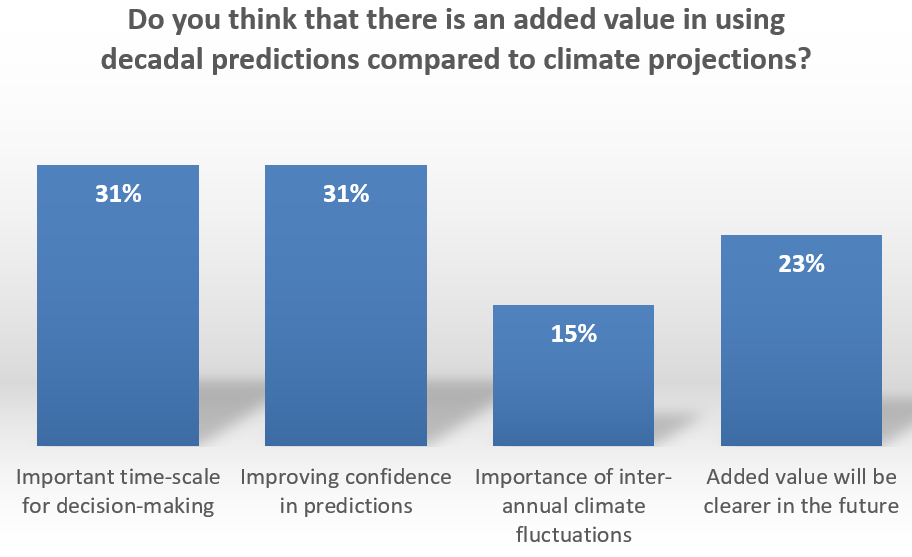


**Figure S8:** Aggregated survey response from the climate scientists on ‘if they think there is an added value in using decadal predictions compared to climate projections?’

**References:**

Bilbao, R., Wild, S., Ortega, P., Acosta-Navarro, J., Arsouze, T., Bretonnière, P. A., et al (2021). Assessment of a full-field initialised decadal climate prediction system with the CMIP6 version of EC-Earth. Earth System Dynamics. <https://doi.org/10.5194/esd-12-173-2021>

Cherchi, A., Fogli, P. G., Lovato, T., Peano, D., Iovino, D., Gualdi, S., et al. (2018). Global Mean Climate and Main Patterns of Variability in the CMCC‐CM2 Coupled Model. Journal of Advances in Modeling Earth Systems. <https://doi.org/10.1029/2018MS001369>

Doblas-Reyes, F. J., Hagedorn, R., and Palmer, T. N. (2005). The rationale behind the success of multi-model ensembles in seasonal forecasting - II. Calibration and combination. Tellus A: Dynamic Meteorology and Oceanography. <https://doi.org/10.3402/tellusa.v57i3.14658>

Hersbach, H., Bell, B., Berrisford, P., Hirahara, S., Horányi, A., Muñoz-Sabater, J., et al. (2020). The ERA5 global reanalysis. Royal Meteorological Society. <https://doi.org/10.1002/qj.3803>

Müller, W., Jungclaus, J., Mauritsen, T., Baehr, J., Bittner, M., Budich, R., et al. (2018). A higher-resolution version of the Max Planck Institute Earth System Model (MPI-ESM 1.2-HR). Journal of Advances in Modeling Earth Systems. <https://doi.org/10.1029/2017MS001217>

Portmann, F.T., Siebert, S. and Döll, P. (2010). MIRCA2000—Global monthly irrigated and rainfed crop areas around the year 2000: A new high‐resolution data set for agricultural and hydrological modeling. Global biogeochemical cycles. <https://doi.org/10.1029/2008GB003435>

Sellar, A. A., Walton, J., Jones, C. G., Wood, R., Luke Abraham, N., Andrejczuk, M., et al. (2020). Implementation of U.K. Earth System Models for CMIP6. Journal of Advances in Modeling Earth Systems. <https://doi.org/10.1029/2019MS001946>

Solaraju-Murali, B., Gonzalez-Reviriego, N., Caron, L.P., Ceglar, A., Toreti, A., Zampieri, M., et al. (2021). Multi-annual prediction of drought and heat stress to support decision making in the wheat sector. npj Climate and Atmospheric Science. <https://doi.org/10.1038/s41612-021-00189-4>

Vicente-Serrano, S. M., Beguería, S., & López-Moreno, J. I. (2010). A Multiscalar Drought Index Sensitive to Global Warming: The Standardized Precipitation Evapotranspiration Index, Journal of Climate. <https://doi.org/10.1175/2009JCLI2909.1>
